# HIV-1 Remission: Accelerating the Path to Permanent HIV-1 Silencing

**DOI:** 10.3390/v15112171

**Published:** 2023-10-28

**Authors:** Danielle E. Lyons, Priti Kumar, Nadia R. Roan, Patricia A. Defechereux, Cedric Feschotte, Ulrike C. Lange, Niren Murthy, Pauline Sameshima, Eric Verdin, Julie A. Ake, Matthew S. Parsons, Avindra Nath, Sara Gianella, Davey M. Smith, Esper G. Kallas, Thomas J. Villa, Richard Strange, Betty Mwesigwa, Robert L. Furler O’Brien, Douglas F. Nixon, Lishomwa C. Ndhlovu, Susana T. Valente, Melanie Ott

**Affiliations:** 1 Gladstone Institute of Virology, Gladstone Institutes, San Francisco, CA 94158, USA; 2 Department of Internal Medicine, Section of Infectious Diseases, Yale University School of Medicine, New Haven, CT 06510, USA; priti.kumar@yale.edu; 3 Department of Urology, University of California San Francisco, San Francisco, CA 94158, USA; 4 Department of Medicine, University of California San Francisco, San Francisco, CA 94158, USA; 5 Department of Molecular Biology and Genetics, Cornell University, Ithaca, NY 14853, USA; 6 Leibniz Institute of Virology, 20251 Hamburg, Germany; 7 Department of Bioengineering, University of California, Berkeley, CA 94720, USA; nmurthy@berkeley.edu; 8 Innovative Genomics Institute, Berkeley, CA 94720, USA; 9 Faculty of Education, Lakehead University, Thunder Bay, ON P7B 5E1, Canada; psameshi@lakeheadu.ca; 10 Buck Institute for Research on Aging, Novato, CA 94945, USA; 11 U.S. Military HIV Research Program, Walter Reed Army Institute of Research, Silver Spring, MD 20910, USAparsonsm@hiv-th.org (M.S.P.); 12 Henry M. Jackson Foundation for the Advancement of Military Medicine, Bethesda, MD 20817, USA; 13 Armed Forces Research Institute of Medical Sciences, Bangkok 10400, Thailand; 14 Section of Infections of the Nervous System, National Institute of Neurological Diseases and Stroke, National Institutes of Health, Bethesda, MD 20824, USA; avindra.nath@nih.gov; 15 Division of Infectious Diseases, Department of Medicine, University of California San Diego, San Diego, CA 92093, USA; 16 Department of Infectious and Parasitic Diseases, University of Sao Paulo, São Paulo 04023-900, Brazil; 17 HOPE Martin Delaney Collaboratory for HIV Cure Research Community Engagement Ambassador, Washinton, DC 20004, USArstrange@rstrange.org (R.S.); 18 National HIV & Aging Advocacy Network, Washington, DC 20004, USA; 19 Research Department, Makerere University Walter Reed Project, Kampala P.O Box 7062, Uganda; 20 Division of Infectious Diseases, Department of Medicine, Weill Cornell Medicine, New York, NY 10021, USA; 21 Department of Immunology and Microbiology, The Herbert Wertheim UF Scripps Institute for Biomedical Innovation and Technology, Jupiter, FL 33458, USA

**Keywords:** HIV-1 cure, latency, transcriptional silencing, HERV

## Abstract

Despite remarkable progress, a cure for HIV-1 infection remains elusive. Rebound competent latent and transcriptionally active reservoir cells persevere despite antiretroviral therapy and rekindle infection due to inefficient proviral silencing. We propose a novel “block-lock-stop” approach, entailing long term durable silencing of viral expression towards an irreversible transcriptionally inactive latent provirus to achieve long term antiretroviral free control of the virus. A graded transformation of remnant HIV-1 in PLWH from persistent into silent to permanently defective proviruses is proposed, emulating and accelerating the natural path that human endogenous retroviruses (HERVs) take over millions of years. This hypothesis was based on research into delineating the mechanisms of HIV-1 latency, lessons from latency reversing agents and advances of Tat inhibitors, as well as expertise in the biology of HERVs. Insights from elite controllers and the availability of advanced genome engineering technologies for the direct excision of remnant virus set the stage for a rapid path to an HIV-1 cure.

Timothy Ray Brown was the first of six individuals currently considered to be cured from HIV-1. All six had cancer and received hematopoietic stem cell transplantations from donors with a homozygous *CCR5*Δ32 mutation. This rendered their new bone marrow-derived immune cells resistant to CCR5-tropic HIV-1. Despite the remarkable outcome offered by this procedure, it is not feasible on a large scale and would be unethical to consider for most people living with HIV-1 (PLWH) given the lower risk and high effectiveness associated with antiretroviral therapy (ART). Also, isolated disruption of *CCR5* could be harmful and reinfection with X4-tropic viruses is possible. The highly investigated “kick-and-kill” (also known as “shock-and-kill”) approach seeks to eliminate the entire HIV-1 reservoir by forcing viral replication of the latent virus with latency-reversing agents, thereby eliminating the infected cell population via antiviral immunity or cytopathic effects. This approach has led to the consideration of anti-HIV-1 therapeutic vaccination, the use of HIV-1-specific broadly neutralizing antibodies and transfusion of cell therapies, including natural killer cells or autologous T cells as cure strategies [1]. While these methods may obviate the use of ART, the main limitations to further advancing these approaches include challenges in inducing robust expression of the entire HIV-1 latent reservoir; toxicity of first-generation latency-reversing agents; risk of T-cell activation with potential for cytokine release, leading to an immune reactivation inflammatory syndrome; and adverse effects of reactivating agents on cytotoxic T cell and natural killer cell functions. Additionally, although antiretroviral therapy effectively lowers plasma HIV RNA to levels below the detection limits of commonly used clinical tests (less than 20 RNA copies/mL), low levels of HIV RNA may persist within tissues, suggesting that ongoing viral transcription occurs within reservoir cells [2,3,4,5]. New and radically different approaches are clearly needed to achieve an HIV-1 cure. 

One such approach is a novel “block-lock-stop” approach that entails the long-term durable silencing of viral expression coupled with permanent transcriptional deactivation of the latent provirus. Such an approach would provide control of viral replication in the absence of antiretroviral therapy. The goal of block-lock-stop is to turn PLWH into people living without HIV-1 (PLWOH). This is achieved through a graded transformation of remnant HIV-1 in PLWH from persistent to silent into permanently defective proviruses, thus emulating and accelerating the natural path that endogenous retroviruses have taken in the genome over millions of years (Figure 1). We define HIV-1 latency as an incomplete state of viral silencing and seek to intensify this state, ultimately irreversibly inactivating the silenced provirus with advanced genome-engineering technologies. The “block-lock-stop” concept diverges significantly from the prevailing strategy that focuses on forced proviral reactivation and the subsequent elimination of reactivated cells with immunological or toxin-based technologies. “Block-lock-stop” addresses important needs specifically in sanctuaries such as the brain where forced viral reactivation is likely dangerous because of the high density of vulnerable neurons and lack of immune clearance. 

Permanent viral silencing through a block-lock-stop approach may be realized by a progressive, new concept, achievable in two steps: (1) Transcriptional silencing by ‘silencing promoting agents’ (SPAs) which could be epigenetic, metabolic or Tat-modulating drugs used in combination to silence HIV-1 proviruses (akin to the silenced state of human endogenous retroviruses (HERVs)). Currently available Tat inhibitors and the inhibitors of host factors have recently been summarized [6,7]. Future development of HIV-1 transcriptional SPAs will build on success with the Tat inhibitor drug didehydro-Cortistatin A (dCA) that inhibits HIV-1 transcriptional elongation and drives viral gene expression into an induced state of persistent latency in vitro at subnanomolar concentrations [8,9]. It is important to selectively suppress HIV-1 transcription without silencing cellular genes, which can be achieved by targeting viral proteins such as Tat, or cellular proteins that have unique functions on the HIV-1 promoter, and the combinatorial use of both. (2) Once silenced, advanced genome engineering technologies will permanently stop viral replication through epigenetic mechanisms or will mutate the provirus (akin to the mutational decay of HERVs). Unlike current practices, this approach does not involve reactivating the provirus and will not target host genes such as CCR5. Such excision or mutation can be achieved using several new genome-engineering technologies (the recombinase Brec1, triplex-forming peptide nucleic acids (PNAs) and CRISPR-base editors (CRISPR-BE)) that avoid deleterious effects of double-stranded DNA breaks (DSB), the repair of which can lead to cell death or cancer [10]. It will be important to develop targeted delivery strategies that can be delivered directly rather than using cell infusion protocols, which are risky and expensive.

Biological precedent for effectively inactivating invading retroviruses exists in the human genome. HERVs make up ~8% of our genomic DNA and have undergone inactivation and mutational decay over the course of evolution [11,12,13]. These elements are often transcriptionally silenced at the chromatin level by the deposition of repressive epigenetic marks such as trimethylation of lysine 9 in histone H3 (H3K9me3) and DNA methylation at CpG sites. These marks are introduced by a dedicated targeting machinery, including KRAB-Zinc Finger Proteins (KZFP) in embryonic and adult somatic cells [14,15]. Since HIV-1 has been recently introduced in humans, a dedicated silencing machinery is lacking, explaining the labile nature of latent HIV-1 and viral replication in the absence of ART.

The HIV-1 promoter has two critical characteristics not present in HERVs: (1) the presence of a paused polymerase complex at the start of transcription, which controls chromatin organization of the viral promoter and keeps the locus epigenetically “open”; and (2) the viral transactivator, Tat, which binds to the TAR RNA element at the 5′ ends of viral transcripts and recruits the host super elongation complex (SEC), overcoming polymerase pausing and driving high-level viral transcription. These unique aspects of HIV-1 transcriptional regulation can be targeted to achieve latency silencing. In particular, didehydro-cortistatin A (dCA) is an attractive SPA target [8,16]. dCA binds to the basic domain of Tat and blocks its binding to the HIV-1 mRNA (TAR), inhibiting its transactivation activity. Over time, Tat inhibition by dCA prompts the viral promoter to enter into a deep state of transcriptional inhibition that is resistant to viral reactivation [16,17]. While the use of early Tat inhibitors was not successful in suppressing active infection and had potential non-specific toxicity, the effectiveness of newer Tat inhibitors in driving latent infection into a silenced state remains to be clinically evaluated [18]. Adding dCA to ART-suppressed humanized mice reduces viral RNA in tissues and significantly delays and diminishes viral rebound upon treatment interruption [19]. However, its complex structure makes dCA very expensive to produce [20]. Efforts for a cost-effective synthesis pathway and cheaper analogs are underway [9]. Additional SPA candidates, structurally distinct from dCA that embody bioequivalent activity and/or targeting other regulatory aspects of HIV-1 transcription, are needed in the pre-clinical pipeline. 

Effective use of the “block-lock-stop” approach will require research into several key areas:

## 1. The Epigenetic Architecture of the Integrated Provirus at Different Integration Sites That Prevents Permanent Silencing of Latent HIV-1

Epigenetic modification of HIV-1 chromatin (e.g., methylation/demethylation) alters its structure to activate or repress transcription [21,22,23]. While repressive histone marks are found at the latent HIV-1 LTR, widespread and stable DNA methylation is lacking [24,25,26,27,28,29,30]. The transcription start site is continuously occupied by a paused polymerase complex, preventing nucleosome repositioning and robust silencing. The key for silencing will be establishing a dense state of chromatin (heterochromatin) around the latent proviruses similar to what is naturally achieved at silenced developmental genes or HERVs and which does not depend on continuing drug treatment. An in-depth examination of host factors that regulate latency in primary CD4^+^ T cells by high-resolution nucleosome mapping and other cutting-edge technologies that are tailored to dissecting transcriptional and epigenetic states (BEM-seq, ChAR-seq, scRNA-seq, scATAC-seq, Ab-seq and PICh) will set the stage for the rational development of small-molecule therapeutics for a “block-lock-stop” strategy. 

## 2. The Cell Types and Epigenetic Cell States That Favor Viral Rebound

Most of the HIV-1 reservoir is in tissues [31] and likely serves as the site of viral recrudescence when ART is interrupted, yet tissues’ reservoirs are understudied relative to blood reservoir. Although methods have been developed to quantitate the reservoir, the basic ability to phenotype cells capable of reactivation has been challenging, specifically in the brain. These cells are rare in vivo and must be stimulated ex vivo for phenotyping since viral proteins are typically not expressed at detectable levels in unstimulated reservoir cells [32,33,34]. Rapid access to freshly deceased tissues and important new technologies can help to address these issues [35,36,37,38,39,40]. These technologies include a dual-fluorescence-labeled virus HIV_GKO_ [28], a Predicted Precursor as determined by SLIDE (PP-SLIDE) analysis approach to define the features of in vivo inducible reservoir cells prior to ex vivo stimulation [41,42], and the identification of CD127 as a surface marker of tissue-resident memory T cells preferentially harboring inducible HIV reservoir cells [43]. 

## 3. The Molecular Functions of Tat and Host Factors That Prevent Permanent Silencing

Durable silencing of HIV-1 transcription by dCA uncovered a key role of Tat in preventing latency. dCA abrogates virtually all virus reactivation from latently infected primary CD4^+^ T cells explanted from PLWH on suppressive ART [8,9]. Nevertheless, a nucleosome-free region remains upstream of the HIV-1 transcription start site under dCA treatment, suggesting that dCA-treated cells are not “irreversibly” silenced [8,9,16,17]. More work is needed to clarify how HIV-1 silencing is controlled at the chromatin level, particularly in response to dCA, and if combining other SPAs with Tat antagonists may help to further silence the HIV-1 promoter. One such potential SPA target is sirtuin-1 (SIRT1), an NAD^+^-dependent lysine deacetylase that deacetylates Tat to enhance its activity. SIRT1 acts as a nutrient sensor and major metabolic controller, helping to shift cells from oxidative phosphorylation to aerobic glycolysis, which increases HIV-1 transcription. Conversely, Tat inhibits SIRT1 enzymatic activity by binding its deacetylase domain [44]. We predict that the early inhibition of SIRT1 by Tat creates metabolic conditions that set the stage for full proviral latency when Tat expression is extinguished.

## 4. HERV Silencing in the Human Genome 

Mammals have long been infected by retroviruses and a fraction of these viruses have entered germ cells and endogenized, passing from parent to offspring. Thus, approximately 8% of the human genome is composed of sequences directly derived from germline infections by retroviruses that accumulated over the past ~100 million years. None of these HERVs are known to be a fully functional retrovirus or capable of new chromosomal integrations; however, most retain noncoding regulatory sequences (generally within their long terminal repeats, LTR) that have the capacity to act upon adjacent genes and dysregulate genome function [14,45]. However, most HERVs are kept transcriptionally silent via a dedicated repressive DNA methylating machinery, including the host protein KAP1 (TRIM28) which is tethered to HERV DNA via sequence-specific KZFPs. DNA methylation is brought to ERVs in part via KRAB-ZFP which recruits de novo methyltransferase DNMT3 via KAP1. KAP1 recruitment leads to an increase in DNA methylation at HERV loci, fostering a heterochromatic epigenetic state [46]. Little is known about HERV silencing in T cells. In human primary CD4^+^ T cells, KAP1 is detected in ~30% of HERVs at specific positions in different HERV families. As KAP1 interacts with repressive KZFPs, this implicates the recruitment of KZFPs as a mechanism silencing HERVs in CD4^+^ T cells [47], but the specific KZFP identity remains unknown. For a thorough summary of ERV DNA methylation and regulatory networks, please see our recent review [46]. Interestingly, HIV-1 infection is known to modulate HERV expression [48,49,50,51,52,53,54,55,56,57], suggesting a crosstalk in the processes that regulate HERVs and HIVs. Thus, there is much to learn about the mechanisms by which HERVs are locked in T cells to develop new approaches to silence HIV-1. The development of new multidomain KZFPs trained to bind the HIV-1 provirus and induce sequence-specific silencing of latent HIV-1 through targeted DNA methylation offers an attractive method for the long-term silencing of HIV-1. Studying the endogenous retroviruses from the past holds promise to yield valuable insight and innovative approaches to durably silence HIV-1 in the future.

## 5. Advanced Gene-Engineering Approaches for the Generation of Defective HIV-1 Proviruses Using Targeted Delivery Systems That Do Not Require the Reactivation of HIV-1

Host gene editing with recombinases and nucleases has focused primarily on *CCR5* and proviral genome excision has generally targeted transcriptionally active viruses or ART suppressed proviruses [58,59,60,61,62,63,64,65,66,67,68,69,70,71,72,73,74]. The genome editing of latent HIV-1 has been accomplished with targeted endonucleases, such as CRISPR/Cas9, zinc finger nucleases, TALENs and site-specific recombinases, which have shown great clinical potential [61,62,63,64,65,66,67,68,75]. There is compelling in vitro data showing that genome-editing enzymes directly target integrated HIV-1 sequences and lead to permanent inactivation of the provirus and, potentially, a ‘classical cure’ for HIV-1 [58,59,60,61,62,63,64,65,66,67,68,75]. Yet, using these approaches for HIV-1 therapy has been challenging, mainly due to suboptimal delivery platforms and a lack of ‘safe’ genome-engineering systems. Current approaches involve transplanting cells after gene editing in cell culture or using adeno-associated viral vectors for delivery in vivo [58,59,69,70,71,72,73,74,75]. These studies provide proof-of-concept in animal models but face translational hurdles from challenges with cell transplant protocols, efficacy and the risk of genotoxicity due to DSBs [76,77].

The development of advanced novel genome-engineering technologies and novel sequence-specific silencing approaches that target both host and viral sequences is needed for an HIV-1 cure. The complete elimination of proviruses throughout the body will be challenging; thus, target host susceptibility genes like *CCR5* would be a synergistic approach that reduces the chances of reinfection. These include the reengineering of Brec1 [59], a traceless recombinase specific for HIV-1 LTR that could be transformed into a new silencing agent. These also include other gene-engineering approaches (e.g., triplex-forming PNAs [78] and CRISPR-derived base editors [Bes] [79]) that target the provirus directly, like Brec1, but avoid DSBs. Many of the current models involve activating the HIV-1 provirus before treatment with genome-engineering technologies, which poses significant risks associated with increased viral loads, especially within the central nervous system where viral reactivation has serious deleterious effects [80,81]. While targeting non-active chromatin for genome engineering is a relatively new field, there is significant evidence for its feasibility [82,83,84,85,86,87]. The significant knowledge about the state of chromatin on the HIV-1 promoter under different conditions makes it an excellent system for the development of genome editing methods that are effective in non-active genes or heterochromatic regions of the genome. Focus on the targeted in vivo delivery of genome engineering therapies rather than cell infusion will also be needed to overcome current delivery challenges. There is significant risk associated with cell infusion methods, which are also prohibitively expensive for many PLWH around the world. Targeted delivery methods will have to be influenced by the identification of HIV-1 reservoirs and the ability to target cell markers that are not unique to active HIV-1. New humanized CD7 antibodies targeting T cells, viral-like particles (VLPs) packaging CRISPR-BE or Brec1 in a trace-less, scarless, non-integrating, DNA-free manner and biocompatible PLGA-nanoparticles for PNA delivery are enticing new areas of research. 

## 6. Community Engagement

It is critical to recognize that novel HIV-1 cure research requires mutual understanding, participation and trust between researchers and the PLWH communities for its ultimate success. HIV-1 cure approaches must be scalable and cost-permissive in low- and middle-income countries. Early partnerships with communities during HIV-1 cure research design allows communities to offer insights into avenues for research at the initial stages of project development. The HOPE Community Engagement Team employs a CAIR (Community Arts Integrated Research) Program and is guided by an Equity Space model and Parallaxic Praxis framework [88] that value and support all stakeholder voices. The framework drives focus group design where participants engage in arts-integrated research activities. The arts serve as a connector and knowledge-generating tool.

Community partners help develop HIV-1 cure curricula that resonate at local levels in diverse countries and among people in historically stigmatized populations [89]. 

The block-lock-stop approach may hold special appeal for HIV long-term survivors since this strategy avoids potential clinical risks inherent in reactivating the latent HIV reservoir and it is feasible (in theory) for PLWH for whom treatment with ART was not initiated until chronic infection, i.e., the majority of PLWH globally. Moreover, some of the Tat and JAK inhibitors that have shown promise for the “Block” phase may also help to reduce chronic immune activation and the resulting systemic inflammation that contributes to advanced/accelerated aging.

## 7. Conclusions

Current ART regimens are highly effective in suppressing HIV-1 replication but do not eliminate the small reservoir of HIV-1 that persists and these drugs must be taken daily for life. Furthermore, ART does not fully reverse immune deficits associated with HIV-1 infection [90], and PLWH on ART can also suffer from chronic inflammation associated with immune activation caused by low-level viral production [91,92,93,94,95] and associated with multiple comorbidities. Thus, there is an urgent need to explore novel therapies that lead to viral eradication or a functional cure. Functional cures which involve the complete suppression of viral gene expression would reduce these risks, similar to elite controllers who have been shown to have low levels of immune activation [96,97,98]. Although “shock and kill” efforts have so far failed to significantly reduce the latent HIV-1 reservoirs, immunotherapeutic approaches in combination with new strategies may be required to achieve durable viral control in the absence of ART [99]. For the “block-lock-stop” approach to work, several questions will need to be answered. Will the silencing be specific to HIV-1 transcription? How long will the silencing be functional? Will gene editing reach all the reservoirs? Although several hurdles remain, the “block-lock-stop” offers a new and under-explored approach to achieve ART-free HIV-1 remission.

## Figures and Tables

**Figure 1:**
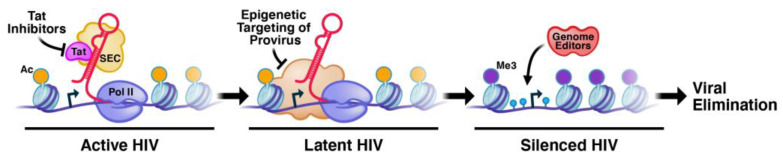
The block-lock-stop approach is multipronged to drive HIV-1 from reversible latency to deep silencing and permanent inactivation.

## Data Availability

No new data were created or analyzed in this study. Data sharing is not applicable to this article.
